# Research priorities in pleural disease: study protocol for a joint patient–provider Delphi consensus statement

**DOI:** 10.1136/bmjresp-2025-003287

**Published:** 2026-04-02

**Authors:** Jane A Shaw, Alice Milne, Federico Mei, Uffe Bodtger, Steven P Walker

**Affiliations:** 1 Stellenbosch University, Stellenbosch, South Africa; 2 Academic Respiratory Unit, University of Bristol, Westbury on Trym, UK; 3 Department of Biomedical Sciences and Public Health, Polytechnic University of Marche, Ancona, Italy; 4 Respiratory Medicine, Nastved Hospital, Næstved, Sjaelland, Denmark; 5 Faculty of Health Sciences, University of Southern Denmark, Odense, Denmark

**Keywords:** Pleural Disease, Mesothelioma

## Abstract

**Introduction:**

There is a wide global variation in research priorities and current clinical practise among pleural medicine practitioners. Research performed today will inform future clinical practice, but the patient’s voices are often not heard in setting the research agenda. The International Multicentre Pleural Research Collaborative (IMPACT) European Respiratory Society (ERS) Clinical Research Collaboration (CRC) aims to present a consensus document from both pleural disease experts and patients, which will guide pleural disease research in the immediate future. The objective is to ensure a focus on scientifically valid, clinically meaningful and patient-centred pleural disease research with global relevance.

**Methods and analysis:**

The core working group will collate a list of previously identified research questions in the key topics in pleural disease: pleural infection, pleural malignancy, pneumothorax, pleural mesothelioma and non-malignant pleural effusions. These questions will be ranked in importance (based on a judgement of scientific merit, significance, innovation, relevance and feasibility) using a Delphi method, by a panel of pleural disease experts. The questions which reach consensus will be subject to a Delphi method survey of patients. The patient-modified list will be discussed in a consensus group meeting involving both experts and patients, who will produce the final prioritised list of research questions.

**Ethics and dissemination:**

Ethical approval will be waived for the active involvement of patients as either advisors or participants in questionnaires. The results will be reported in the form of a peer-reviewed publication with an open access license, according to the ACCORD (ACcurate COnsensus Reporting Document) guidelines for consensus-based research.

**WHAT IS ALREADY KNOWN ON THIS TOPIC**

- Research priorities of global pleural medicine experts are yet to be aligned, and the priorities of patients have not been assessed in setting the research agenda.

**WHAT THIS STUDY ADDS**

- This study will produce a set of ranked research questions for pleural medicine that have been agreed on through discussion between expert clinicians and patients with pleural disease.

**HOW THIS STUDY MIGHT AFFECT RESEARCH, PRACTICE OR POLICY**

- This study will guide future research in the field of pleural medicine.

## Introduction

 Pleural disease is a broad and heterogeneous field of medicine which encompasses infection, malignancy, effusion and pneumothorax. The incidence of all pleural diseases is expected to increase over time as the number of people with risk factors for pleural disease grows, including the elderly, people living with chronic disease, surviving cancer or severe heart failure.^1^ The field of pleural medicine is also developing rapidly, with an increased availability of bedside thoracic ultrasound and many pleural-relevant publications based on practice-changing randomised controlled trials.^2^,^4^ Research interest and funding opportunities are increasing as the field develops; therefore, there is a need to define the research agenda, particularly from the perspective of the patients who suffer from these diseases.

The International Multicentre Pleural Research Collaborative (IMPACT) is a European Respiratory Society (ERS) Clinical Research Collaboration (CRC) which consists of over 200 clinicians in more than 50 countries who are either experts in pleural disease or have a special interest in this field. Interactions within the IMPACT network have highlighted the heterogeneity of current practices and the need to better understand research priorities, even among those who are considered pleural disease experts. Furthermore, there has been an evolution in the outcomes used in pleural intervention research, towards more patient-focussed measures of success. Studies which have used these patient-focussed tools have triggered a marked change in clinical practice, highlighting the importance of understanding our field from the perspective of those who suffer the illness in question.^5 6^

The field of pleural medicine needs a unified set of objectives, which can drive research, and consequently, influence global clinical practice. Therefore, we will perform a formal Delphi survey of expert clinicians in pleural medicine using existing research questions identified in guideline documents. Most importantly, patients will contribute to the pleural disease research agenda and guide clinicians in pursuing objectives which are clinically meaningful.

## Methods and analysis

**Figure 1:**
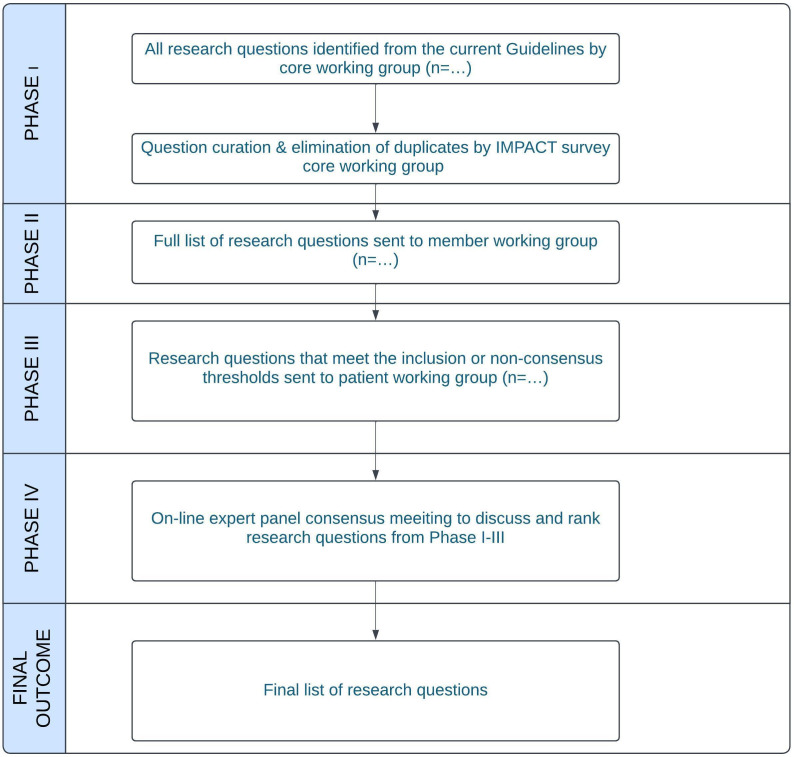
The Delphi consensus process.

We will use a Delphi approach to generate a consensus document which lists the current priority research questions for pleural disease, according to global pleural disease experts and patients. There will be four phases of work (figure 1).

### Phase I

A core working group will evaluate the available international guidelines on key topics in pleural disease to identify research recommendations endorsed by international guideline committees in the field of pleural disease.^7^,^14^ This list will be curated and condensed to remove repetitions, discrepancies or inconsistencies. The key topics will include pleural infection, pleural malignancy, pneumothorax, pleural mesothelioma and non-malignant pleural effusions.

### Phase II

A members working group composed of at least 20 IMPACT ERS CRC members will be established. These selected members will be international experts in pleural disease and include members of the IMPACT International Scientific Advisory Board. They will be selected to ensure geographic representation, early career representation and gender representation. Where a selected IMPACT member is invited but declines to contribute, another member with similar expertise level and demographics will be invited in their place to maintain the balance within the group.

Once the members’ working group is finalised, a short online meeting will be held during which the Delphi process will be explained. Thereafter, participants will be sent a link to the online survey using commercially available Delphi survey management software. The survey will be preceded by a cover page which explains the process again, and each new section will have the same preceding explanatory text. The electronic survey will be developed and tested by the core working group, and blind-tested by at least one person outside of the working group, before it is sent to the members for responses. Members will be asked to rank the importance of each candidate research question, grouped according to each key pleural disease topic, individually on a 9-point Likert scale, considering five criteria: scientific merit, significance, innovation, relevance and feasibility. After each research question ranking, there will be an optional free text comment box, where participants can clarify, modify or add to research questions.

The core working group will employ several reminders, leveraging pre-existing IMPACT network communication channels to maximise the response rate during the Delphi process and try to minimise responder fatigue and drop-out. Because of the small number of members in the survey working group, a minimum of 80% responses to the survey will be required to finish the round. Once all the responses to the first round have been received, the core working group will compile the rankings for each research question and any additional responses. Using predetermined consensus thresholds (table 1), questions will either be included in phase III, excluded or subject to a second round of rankings by the members.

**Table 1:** Phase II consensus thresholds

Inclusion	>75% of respondents provide a positive result (five or above) on the Likert scale for all criteria.
Exclusion	>75% of respondents provide a negative result (one to four) on the Likert scale for all criteria.
Non-consensus	When the proposed priority research question has met neither the inclusion nor exclusion consensus thresholds.

The second round of the survey will be preceded by a cover page with general feedback from the first round (number of questions which reached consensus for inclusion or exclusion, and number which did not reach consensus) and instructions for the second round. Each key topic section of research questions will be preceded by detailed feedback on the responses to those questions in the first round, including the free-text comments, to allow respondents to modify their previous responses and move towards a consensus.

Research questions that meet the non-consensus thresholds in the second Delphi round will be subjected to a third and final round of rankings, also preceded by detailed feedback from the previous round. Research questions that meet the exclusion consensus threshold will not be brought forward for review. If none of the research questions meet the inclusion or non-consensus thresholds, the thresholds will be lowered until a critical mass of research questions is able to be brought forward to the next phase. We consider a priori that a critical mass of 20 priority research questions will suffice. The core working group will conduct a maximum of three Delphi rounds.

### Phase III

Phase III will involve the core working group and a patient working group which will be developed in collaboration with the European Lung Foundation (ELF). Patients from each key disease area (pleural infection, pleural malignancy, pneumothorax and pleural mesothelioma, and non-malignant pleural effusions) will be invited to participate in the disease-specific survey section. Research questions that meet the inclusion criteria or consensus threshold at end of phase II will be translated into language accessible to patients. A short description of the background to each research question on the list, in non-scientific language, will be provided. The patient working group will then be asked to rank each candidate research question individually on a 9-point Likert scale for two criteria: importance and patient focus. A space for free-text commentary on the research questions for their topic will appear at the end of the questions. We will aim for at least 100 patient questionnaire responses, with a minimum of 25 in each patient group. We will use purposeful sampling to identify a representative sample of patients who have experienced a phenomenon of interest. We will work with ELF to develop and share the questionnaire in multiple international countries, represented by the IMPACT group. Patient groups and charities will be approached, with the assistance of ELF. Members of the IMPACT collaborative will also approach outpatients and inpatients. Language will be tested both with local members and patients. Efforts will be made to ensure that there are representative responses from low-income and middle-income countries. Local IMPACT representatives and patient groups will check the language. Disagreements will be discussed in phase IV (panel meeting).

Research questions that meet the inclusion thresholds will progress to phase IV. Research questions that meet the exclusion consensus threshold will not be brought forward for review. If none of the research questions meet the inclusion, the thresholds will be lowered until a critical mass of research questions is able to be brought forward to the next phase. We consider a priori that a critical mass of 10 priority research questions will suffice. However, Delphi processes tend to be flexible and, therefore, it is more likely that there will be a large number of questions brought forward for discussion. If this is the case, we will rank the questions in tiers and give priority to discussion of the highest-ranking tier.

### Phase IV

Phase IV will be conducted in an online expert panel meeting to generate consensus. An experienced facilitator will moderate the meeting. We will invite all participants from phases I, II and III to participate, as well as representatives from patient advocacy groups. Should too many individuals express their interest in participating on the expert panel, we will select a diverse and balanced group comprised of 20 individuals, with at least one individual from each of the geographical, stakeholder and career stage groups.

This meeting will include:

1.  Overview of Delphi methodology.
2.  Overview of results from phase I to III.
3.  Discussion of consensus questions.
4.  Discussion of non-consensus questions.
5.  Anonymous scoring of questions.
6.  Compilation of the final ranked list of research priorities.

## Data collection and handling

Data will be collected electronically and stored in two-factor authentication-protected cloud-based storage, hosted by one of the core working group member’s academic institutions, with all associated protections from data loss and theft. Only members of the core working group will have access to the data. When the results have been analysed and reported, data will be fully deidentified and locked to prohibit editing.

The surveys will be conducted by Delphi management software with inherent safety protocols and code-based reporting tools.

## Ethics approval

The Delphi survey will be conducted in accordance with the principles of the Declaration of Helsinki and Good Clinical Practice. The participants’ responses to the Delphi survey will be kept anonymous to each other and identifiable only to the core working group. Ethical approval will be waived for the active involvement of patients as either advisors or participants in questionnaires.

## Dissemination

The results will be reported in the form of a peer-reviewed publication with an open access license, according to the ACCORD (ACcurate COnsensus Reporting Document) guidelines for consensus-based research.^15^ We will use the GRIPP2 (Guidance for Reporting Involvement of Patients and the Public) reporting checklist to report the findings transparently.^16^ Results will also be presented at the annual IMPACT CRC meeting and the ERS Congress to ensure wide dissemination. Lastly, results will be posted on the IMPACT website. The final research questions, as an output of a robust prioritisation process, may support funding applications and help attract and advocate for increased investment in pleural disease research.

## Data Availability

No data are available.
